# Amoxycillin and Metronidazole Therapy for Helicobacter pylori Eradication: A 10-Year Trend in Turin, Italy

**DOI:** 10.4274/balkanmedj.2015.1714

**Published:** 2017-05-15

**Authors:** Davide Giuseppe Ribaldone, Marco Astegiano, Giorgio Saracco, Rinaldo Pellicano

**Affiliations:** 1 Gastroenterology Unit, S. Giovanni Battista (Molinette) Hospital, Turin, Italy; 2 Department of Oncology, University of Turin, Turin, Italy

## To The Editor,

*Helicobacter pylori (H. pylori)* infection plays a crucial role in causing gastritis and peptic ulcer disease (PUD). To date, despite the several regimens proposed, no therapy leads to a 100% *H. pylori* eradication rate (1). As the antimicrobial activity of metronidazole is marginally affected by low pH, this drug may be highly effective against *H. pylori*. The European Helicobacter Study Group has advised to employ a metronidazole-based triple therapy as choice in treating *H. pylori* infection in relation to specific resistance rate in each region (2). Recently, a multicentric study revealed that, in Southern Europe, the primary rate of *H. pylori* metronidazole resistance was around 30% (3).

In 2002, we found that in Turin, Northern Italy, a triple therapy with metronidazole, amoxycillin and a proton pump inhibitor (PPI) for 7 days or for 10 days, achieved an eradication rate of 73.5% or 71.6%, respectively. Lengthening the treatment conferred no advantages. These values were significantly lower compared to those reported earlier (4). In a subsequent study with alternative schedules (cefixime plus metronidazole), the results did not improve (5). As antibiotic resistance is an evolving process, it is mandatory to carry out surveys in order to be guided in the therapeutic choice. Hence, in the year 2013 (January 01 - December 31), we evaluated prospectively the *H. pylori* eradication rate of consecutive naive patients, treated with a triple therapy comprising a standard dose of PPI, amoxycillin 1 g and metronidazole 500 mg twice daily. Results were compared with a previous randomized prospective study conducted 10 years earlier with the same schedule (4). Eradication of *H. pylori* infection was assessed by ^13^C-urea breath test, performed according to the supplier’s instructions (Helicobacter Test, INFAI^®^, Bochum, Germany). The reported sensitivity is 97.9% and specificity 98.5%. Samples were analysed for ^13^C/^12^C ratio with a mass spectrometer (BreathMAT plus, Finnigan, Bremen, Germany). Results were expressed as excess ð^13^CO_2_ excretion per mil: a value >4 delta per mil was considered positive. No patient had received PPI or antibiotics in the last 30 days. All patients gave their written informed consent. Differences in eradication rates were tested with the chi-square test (with Yates’ correction for continuity). A p value <0.05 was considered significant. The cohort included 66 patients (40 males, mean age 61.6, range 39-62), 39 of them received a regimen including a 1-week triple therapy (group I) and 27 were treated with a 10-day triple therapy (group II). Forty-two of them had a previous diagnosis of PUD or gastroduodenal erosions, and the remaining of active gastritis. The overall *H. pylori* eradication rate was 69.2% (27/39) in group I and 70% (19/27) in group II, without significant difference (p=0.96). When compared with the prospective study published in the year 2002 (4) no differences were observed in the effectiveness of therapy (p=0.81 for 7 days and p=0.95 for 10 days) (Table 1). In conclusion, in our area, a metronidazole-based treatment regimen for *H. pylori* eradication, although unsatisfactory, is as effective as 10 years ago.

## Figures and Tables

**Table 1:**
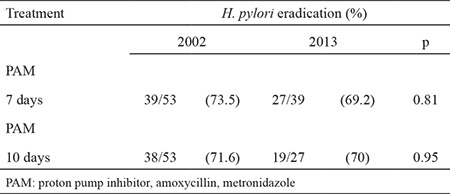
Results of H. pylori eradication regimens
